# The nuclear corepressor 1 and the thyroid hormone receptor β suppress breast tumor lymphangiogenesis

**DOI:** 10.18632/oncotarget.12978

**Published:** 2016-10-27

**Authors:** Olaia Martínez-Iglesias, David Olmeda, Elvira Alonso-Merino, Sara Gómez-Rey, Ana M. González-López, Enrique Luengo, María S. Soengas, José Palacios, Javier Regadera, Ana Aranda

**Affiliations:** ^1^ Instituto de Investigaciones Biomédicas “Alberto Sols”, Consejo Superior de Investigaciones Científicas and Universidad Autónoma de Madrid, Spain; ^2^ Molecular Oncology Programme, Centro Nacional de Investigaciones Oncológicas, Universidad Autónoma de Madrid, Spain; ^3^ Departamento de Anatomía Patológica, Hospital Universitario Ramón y Cajal, Instituto de Investigación Sanitaria Ramón y Cajal (IRYCIS), Universidad de Alcalá, Spain; ^4^ Departamento de Anatomía, Histología y Neurociencia, Facultad de Medicina, Universidad Autónoma de Madrid, Spain

**Keywords:** thyroid hormone receptor beta 1, nuclear receptor corepressor 1, lymphangiogenesis, VEGFs, breast cancer

## Abstract

Vascular Endotelial Growth Factors C and D (VEGF-C and VEGF-D) are crucial regulators of lymphangiogenesis, a main event in the metastatic spread of breast cancer tumors. Although inhibition of lymphangiogenic gene expression might be a useful therapeutic strategy to restrict the progression of cancer, the factors involved in the transcriptional repression of these genes are still unknown. We have previously shown that Nuclear Receptor Corepressor 1 (NCoR) and the thyroid hormone receptor β1 (TRβ) inhibit tumor invasion. Here we show that these molecules repress *VEGF-C* and *VEGF-D* gene transcription in breast cancer cells, reducing lymphatic vessel density and sentinel lymph node invasion in tumor xenografts. The clinical significance of these results is stressed by the finding that NCoR and TRβ transcripts correlate negatively with those of the lymphangiogenic genes and the lymphatic vessel marker *LYVE-1* in human breast tumors. Our results point to the use of NCoR and TRβ as potential biomarkers for diagnosis or prognosis in breast cancer and suggest that further studies of these molecules as potential targets for anti-lymphangiogenic therapy are warranted.

## INTRODUCTION

Metastasis is the main cause of cancer-related deaths. Although some malignant tumors metastasize via the bloodstream most epithelial cancers, including breast tumors, first spread via lymphatic vessels to their regional lymph nodes and indeed the detection of tumor cells within the sentinel node has a main importance for patient prognosis [1, 2]. Expression of the lymphangiogenic growth factors by the tumor cells induces lymphangiogenesis, the growth and enlargement of lymphatic vessels, playing a crucial role in tumor dissemination [3–6]. Tumor lymphangiogenesis is mostly due to the proliferation and sprouting of pre-existing vessels, rather than to incorporation of circulating endothelial progenitor cells and is at least in part mediated by VEGF-C and to a lesser extent VEGF-D [3, 5, 7–11]. Lymph vessel density correlates with nodal status and is a prognostic factor in breast cancer [7, 12–15]. Tumor-associated macrophages can also produce lymphangiogenic factors contributing to vessels formation [16], thus showing the importance of the tumor microenvironment in this process. In addition, the lymphatic endothelial cells produce chemokines such as the stromal-derived factor 1 (or CXCL12), which bind CXCR4 receptors in the tumor cells [17, 18], facilitating their migration toward the lymphatic vessel [19].

NCoR (or Nuclear Corepressor-1) plays an important role in gene silencing. This corepressor associates with histone deacetylases (HDACs) and is recruited to target genes by interaction with nuclear receptors and other transcription factors, causing chromatin compaction and blocking transcription [20–23]. Through regulation of gene expression this corepressor could modulate cancer cell biology. Indeed, NCoR mutations have been found in breast tumors, and in these tumors frame-shift or nonsense inactivating mutations of the NCoR gene have been identified as driver mutations [24–26]. These observations support the findings that low NCoR expression is associated with invasive breast tumors [27, 28], a shorter relapse-free survival [29] and resistance to anti-estrogen treatment [30], suggesting the role of NCoR as a tumor suppressor. In agreement with this hypothesis, NCoR silences transcription of genes associated with metastatic growth and poor outcome in cancer patients, inhibiting tumor growth, invasion and metastatic potential in xenograft mouse models [31]. Furthermore, it has been demonstrated the existence of a positive auto-regulatory loop that maintains *NCoR* gene expression, suggesting that loss of NCoR expression can confer an advantage to the tumor cell, contributing to tumor progression even in the absence of *NCoR* gene mutations.

The actions of the thyroid hormones thyroxine (T4) and triiodothyronine (T3) are mediated by binding to the nuclear thyroid hormone receptors (TRs). Although the thyroid gland produces more T4, T3 is formed by deiodination of T4 in extrathyroidal tissues and is believed to be the active hormone since TRs show a higher binding affinity for T3 than for T4 [32]. TRs and particularly the TRβ isoform can have tumor suppressor actions. TRβ mutations, anomalous subcellular localization and biallelic inactivation of this gene by promoter methylation has been found in breast tumors [33–35]. Furthermore, expression of TRβ in breast cancer cells reduces tumor growth [36, 37]and has a strong inhibitory effect on invasion, extravasation, and metastasis formation in immunodeficient mice [38]. TRβ induces NCoR expression and this induction appears to be an essential mediator of the tumor suppressive and anti-metastatic actions of the receptor. Moreover, both NCoR and TRβ are downregulated in the more aggressive human estrogen receptor negative (ER^−^) breast tumors with respect to the ER^+^ tumors with a better prognosis, existing a positive correlation between transcript levels of the receptor and the corepressor [31].

In this work we tested the possibility that NCoR and TRβ could regulate the expression of *VEGF* genes and the growth of lymphatic vessels, thus regulating tumor invasion. We demonstrate that NCoR and TRβ repress transcription of the *VEGF-C* and *VEGF-D* genes in breast cancer cell lines and tumor xenografts. Furthermore, NCoR depletion increases lymph vessel density in the tumors and reverses the inhibitory effect of the receptor in lymphangiogenesis. The importance of our results is supported by the finding of a strong negative correlation between the mRNA levels of the lympangiogenic genes and either *NCoR* or *TRβ* in human breast tumors. This correlation is independent of the ER status, although lymphangiogenic genes are expressed at significantly higher levels in the ER^−^ tumors. Since high VEGF-C and VEGF-D levels lead to a poor prognosis in breast cancer, our finding that NCoR and TRβ are potent inhibitors of these factors suggests that they may serve as novel therapeutic targets to inhibit lymphangiogenesis and breast tumor progression.

## RESULTS

### NCoR represses transcription of the VEGF-C and VEGF-D genes in breast cancer cell lines

**Figure 1:**
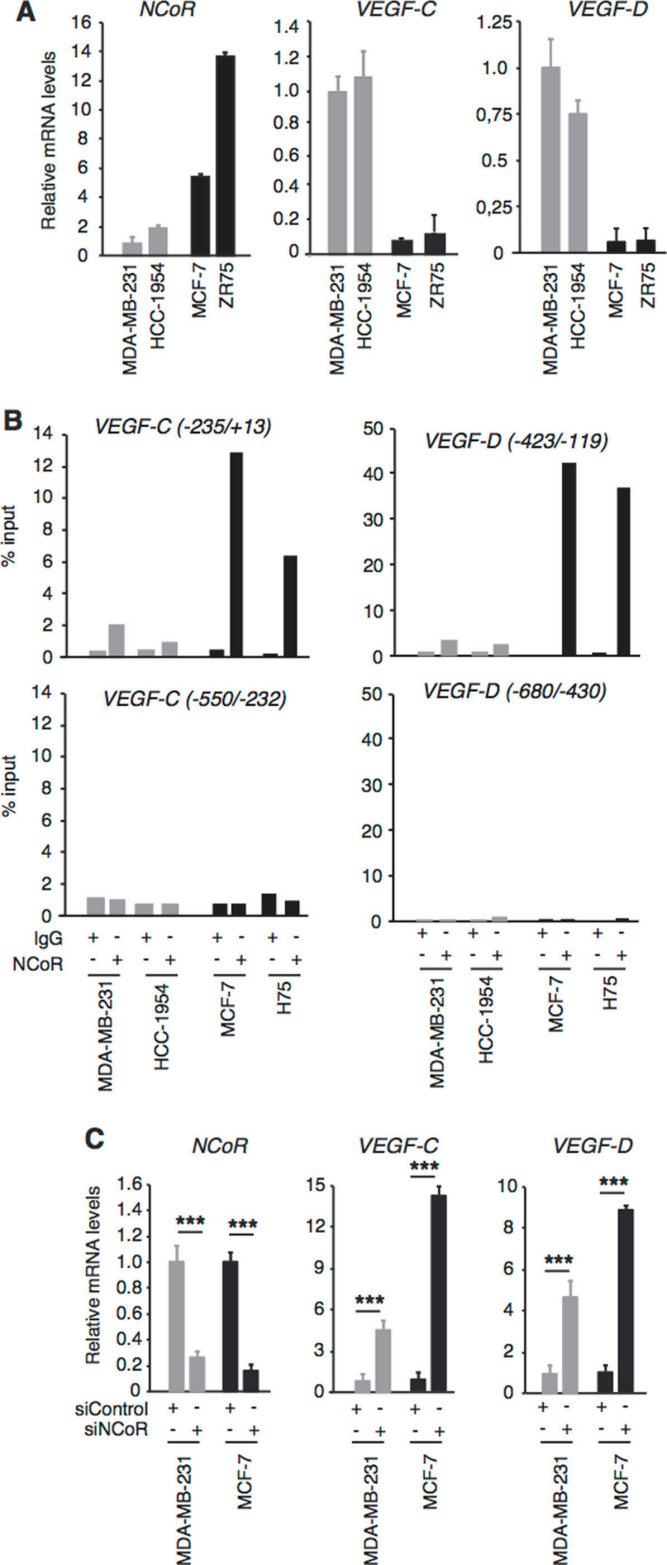
NCoR represses VEGF-C and VEGF-D gene transcription NCoR represses *VEGF-C* and *VEGF-D* gene transcription (**A**) *NCoR*, *VEGF-C* and *VEGF-D* mRNA levels (means ± S.D) were measured by quantitative real-time PCR in the indicated human breast cancer cells lines and are expressed relative to the values obtained in MDA-MB-231 cells. (**B**) chromatin immunoprecipitation (ChIP) assays with NCoR antibody and control IgG were performed with the indicated fragments of the *VEGF-C* and *VEGF-D* gene promoters. Results obtained in two different experiments are shown. (**C**) levels of the indicated transcripts were determined in cells transfected with control or NCoR siRNAs 72 h before. Data (means ± SD) are expressed relative to the values obtained in cells transfected with siControl. Significance of *t*-test between cells transfected with siControl and siNCoR are indicated. ***P < 0.001.

To analyze a possible correlation between *NCoR* and *VEGF-C* and *VEGF-D* gene expression, we first measured their transcript levels in several human breast cancer cell lines, both ER^+^ and ER^−^. *NCoR* mRNA was significantly higher in the ER^+^ MCF-7 and ZR75–1 cells than in the very aggressive HCC-1954 cells and in the MDA-MB-231 cells, while *VEGF-C* and *VEGF-D* transcripts exhibited an opposite pattern, being lower in the ER^+^ positive cell lines (Figure 1A). Although other factors different from NCoR could be responsible for the negative association with lympangiogenic gene expression in these independently-derived cell lines, the inverse relationship observed suggested that NCoR could repress *VEGF-C* and *VEGF-D* gene transcription. Proximal promoter sequences appear to play an important role in the control of *VEGF-C* and *VEGF-D* transcription [39, 40]. To analyze if NCoR could bind to the regulatory region of these lymphangiogenic genes, we performed chromatin immunoprecipitation (ChIP) assays with an NCoR antibody and two different fragments of the 5′-flanking regions of these genes. Significant NCoR association to the −235/+13 region of the *VEGF-C* gene was observed in MCF-7 and ZR75-1 cells, while NCoR binding was much lower in MDA-MB-231 and HCC-1954 cells (Figure 1B). However, NCoR did not bind to the immediate upstream region of the *VEGF-C* promoter in any cell line. A very strong binding of the corepressor was found in the ER+ cells when the proximal −423/−119 region of the *VEGF-D* gene was analyzed and again NCoR binding to these sequences was very low in the ER^−^ cells. However, NCoR was not recruited to the −608/−430 region of the *VEGF-D* gene, previously proposed to be necessary for *VEFG-D* transcription [39, 40] (Figure 1B).

To study the functionality of NCoR binding to the regulatory region of the lymphangiogenic genes, MDA-MB-231 and MCF-7 cells were transfected with a control siRNA or with an NCoR specific siRNA. Transfection of siNCoR very effectively reduced NCoR transcripts in the cells, and *VEGF-C* and *VEGF-D* gene expression was significantly increased upon NCoR depletion (Figure 1C). In accordance with the different levels of NCoR expression and promoter occupancy shown in panels A and B, this increase was stronger in MCF-7 cells and more moderate in MDA-MB-231 cells. These results show that the *VEGF-C* and *VEGF-D* genes are bona fide targets of NCoR in breast cancer cells.

### TRβ silences VEGF-C and VEGF-D gene transcription

**Figure 2:**
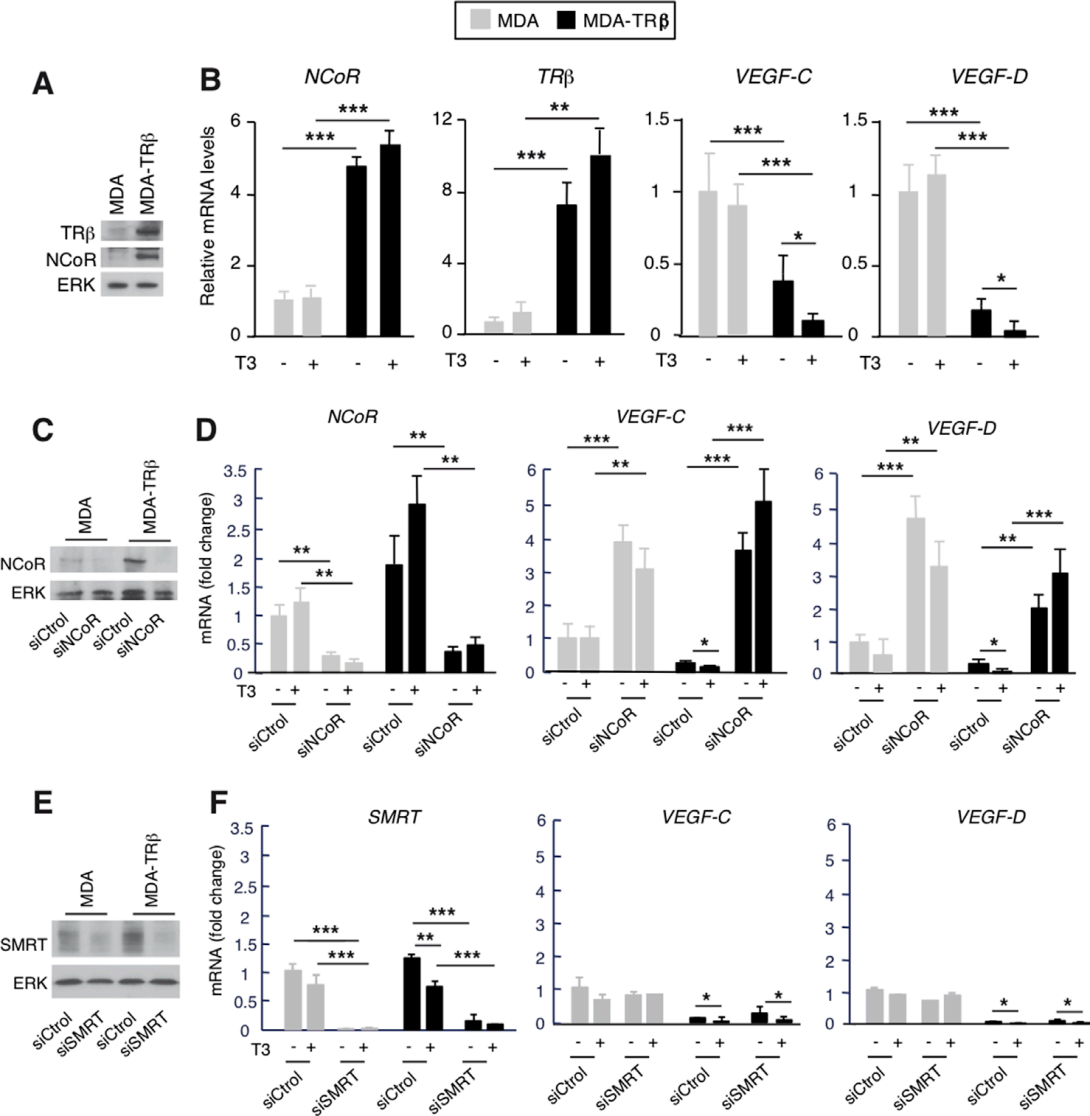
NCoR depletion increases VEGF-C and VEGF-D gene expression (**A**) Western blot analysis of TRβ and NCoR in parental MDA-MB-231 cells and in cells stably expressing the receptor (MDA and MDA-TRβ, respectively). ERK was used as a loading control. (**B**) mRNA levels of the indicated genes were determined in cells treated in the presence and absence of 5 nM T3 for 36 h. (**C**) NCoR and ERK levels after 72 h of transfection with siControl or siNCoR. (**D**) Transcript levels of *NCoR*, *VEGF-C* and *VEGF-D* in cells transfected with siControl or siNCoR and treated with and without T3. (**E**) SMRT and ERK levels after 72 h of transfection with siControl or siSMRT. (**F**) Transcript levels of *SMRT*, *VEGF-C* and *VEGF-D* in cells transfected with siControl or siSMRT and treated with and without T3. All data are means ± S.D and are expressed relative to the values obtained in untreated parental cells transfected with the control siRNA. Significance of ANOVA pos*t*-test among the indicated groups is shown as * *P* < 0.05, **P < 0.01 and ***P < 0.001.

**Figure 3:**
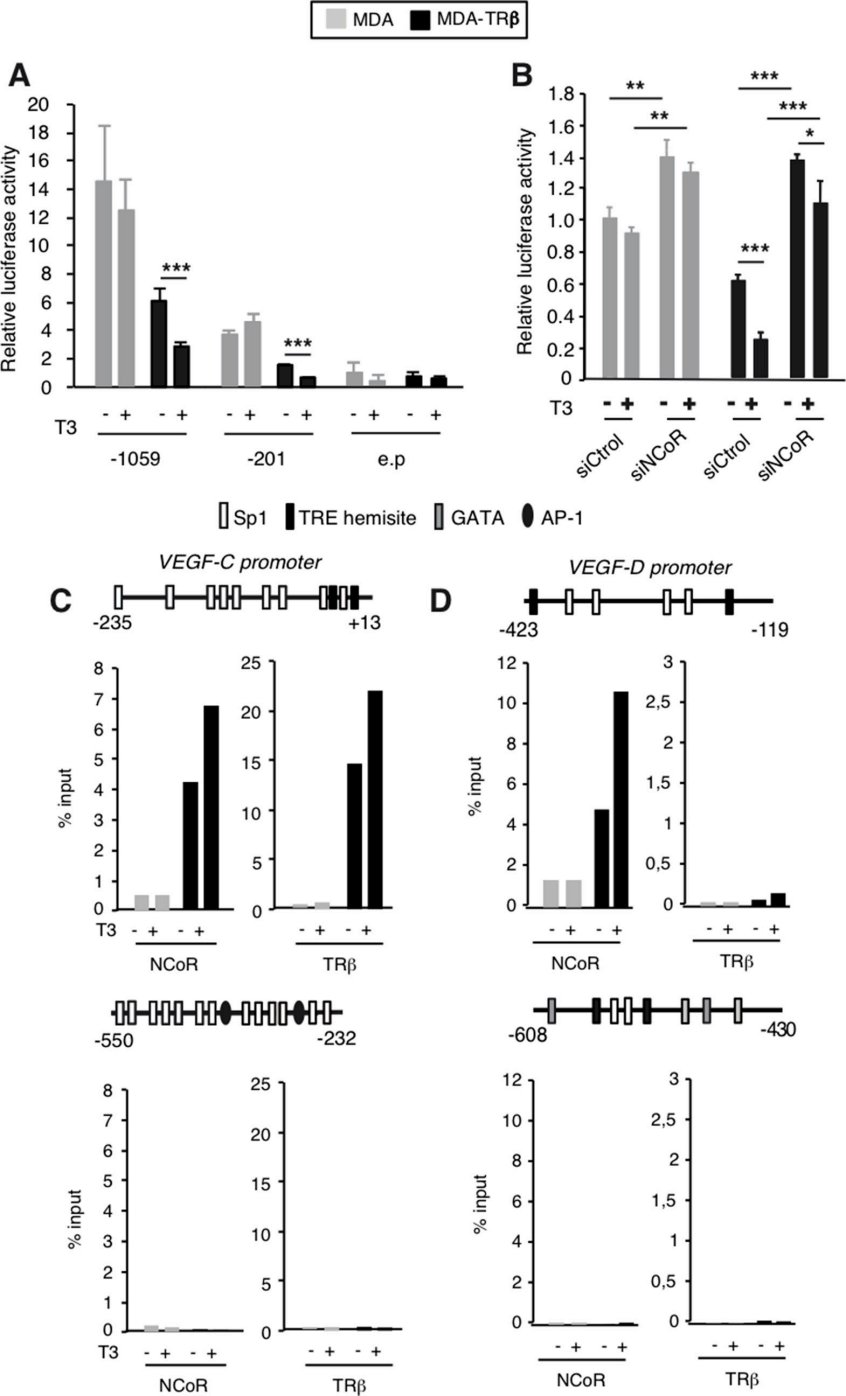
NCoR is involved in transcriptional inhibition of the *VEGF-C* and *VEGF-D* genes by TRβ (**A**) Transient transfection assays in MDA and MDA-TRβ cells with reporter plasmids of the human *VEGF-C* promoter extending to nucleotides −1059 and −201 or the empty plasmid without promoter sequences (e.p). Luciferase activity (means ± S.D) was determined in cells treated for 36 h in the presence and absence of 5 nM T3 and is expressed relative to value obtained in the untreated cells transfected with the empty plasmid. Differences between untreated and T3-treated cells were calculated with the *t*-test and are indicated as ***P < 0.001. (**B**) similar experiments in cells cotransfected with the −201 plasmid and control or NCoR siRNAs. Luciferase activity (means ± S.D) was measured in cells treated with and without T3 and is expressed relative to that obtained in untreated MDA cells transfected with siControl. Statistically significant differences of the ANOVA pos*t*-test among groups of MDA and MDA-TRβ cells are indicated as *P < 0.05, **P < 0.01 and ***P < 0.001. (**C**) ChIP assays with the indicated fragments of the *VEGF-C* and *VEGF-D* promoters and NCoR and TRβ antibodies in cells treated in the presence and absence of T3 for 1 h. Schemes of the promoter fragments used showing the existence of putative binding sites for TR (TRE hemisites) and for other transcription factors are depicted. Results are expressed as % of the input after subtracting the values obtained after immunoprecipitation with control IgG that were always lower than 1% of the input. Data shown are the mean of two independent experiments.

Since TRβ can increase NCoR mRNA and protein levels [31] and this corepressor silences *VEGF-C* and *VEGF-D* gene expression, we next examined the possibility that TRβ could reduce the expression of lymphangiogenic genes and the potential role of NCoR in this repression. With this purpose we first compared *VEGF-C* and *VEGF-D* transcripts in parental MDA-MB-231 cells and in cells expressing TRβ in a stable manner (from now on MDA and MDA-TRβ cells, respectively). As expected from the induction of NCoR expression by the receptor observed in several cell types [31], MDA-TRβ cells expressed higher NCoR protein and mRNA levels than the parental cells (Figure 2A and 2B), and also showed significantly lower levels of *VEGF-C* and *VEGF-D* transcripts. Silencing was observed in the absence of ligand, but incubation with T3 further reduced mRNA levels of lymphangiogenic genes (Figure 2B), showing the role of NCoR and TRβ as inhibitors of VEGFs gene expression in these cells. To analyze the role of NCoR in the repressive effect of TRβ, we next examined *VEGF-C* and *VEGF-D* mRNA levels in MDA and MDA-TRβ cells transfected with siControl or siNCoR (Figure 2C). NCoR depletion increased *VEGF-C* and *VEGF-D* transcripts both in parental and MDA-TRβ cells, strongly relieving the repressive effect of the unliganded TRβ and abolishing the inhibitory effect of T3 (Figure 2D). Therefore, NCoR appears to play a major role in lymphangiogenic gene silencing by TRβ. Since NCoR2 (or SMRT) could have redundant effects with NCoR in transcriptional repression, we conducted similar experiments in cells transfected with a specific SMRT siRNA. In contrast with NCoR, selective SMRT depletion (Figure 2E) did not increase *VEGF-C* and *VEGF-D* transcripts in MDA cells and was unable to relieve the inhibitory effects of TRβ in MDA-TRβ cells (Figure 2F), showing that SMRT does not participate in regulation of the lymphangiogenic genes in these cells. To further investigate the mechanism by which NCoR and TRβ regulate transcription of prometastatic genes, we conducted transient transfection studies with luciferase constructs containing the 5′-flanking region of the *VEFG-C* gene [39] in MDA and MDA-TRβ cells. As shown in Figure 3A, activity of the −1059/+206 promoter region was lower in the TRβ expressing cells and was further reduced in the presence of T3. Similar results were obtained with a shorter construct (−201/+206). However, no changes were observed when cells were transfected with the luciferase plasmid alone, indicating that the proximal promoter region that binds NCoR in ChIP assays also contains the response elements responsible for repression of *VEGF-C* gene transcription by TRβ. NCoR knock-down in cells transfected with the −201/+206 plasmid increased promoter activity in the parental cells, and almost totally reversed the inhibition by TRβ and T3 (Figure 3B), recapitulating the results obtained with the endogenous transcripts in Figure 2 and demonstrating again the important role of NCoR in *VEGF-C* gene silencing. *In silico* analysis of the proximal *VEGF-C* promoter sequences −231/+13 used in the ChIP assays revealed the existence of two putative hemisites that could bind the thyroid hormone receptor. ChIP assays with this region demonstrated that TRβ expression increased remarkably the association of NCoR with the *VEGF-C* gene and that this association was enhanced in T3-treated cells. TRβ also bound constitutively to the same region in MDA-TRβ cells and T3 further increased receptor recruitment to the promoter (Figure 3C). These results correlated with the lower transcriptional activity of the gene under these conditions. The more upstream −550/−232 sequences recruited neither NCoR nor TRβ, discarding their participation in repression by TRβ and confirming that the more proximal promoter sequences identified in the transient transfection assays appear to be sufficient to mediate gene repression by TRβ. Examination of he *VEGF-D* promoter region −423/−119, which binds NCoR, also predicted the existence of two hemisites that could act as binding motifs for TR (Figure 3D). Although NCoR bound strongly to this promoter fragment in MDA-TRβ cells and T3 increased this binding, the receptor was absent from this region. This result indicates that the hemisites do not act as TR binding sequences and that therefore NCoR is not recruited to this region by interaction with TRβ. In addition, the −608/−430 region of the *VEGF-D* gene has been described to contain and atypical hormone response element which could bind orphan nuclear receptors [40]. However, TRβ or NCoR association with this region was not observed in either MDA or MDA-TRβ cells, indicating that this region does not play a role in *VEGF-D* silencing by the corepressor.

### NCoR and TRβ repress VEGF-C and VEGF-D expression in ER^+^ MCF-7 cells

To analyze if the silencing effect of TRβ in lymphangiogenic gene expression was restricted to the MDA cells or could be extended to other breast cancer cells, we next used MCF-7 cells stably expressing high levels of TRβ (MCF7-TRβ cells) [37] (Supplementary Figure S1A). As shown in Supplementary Figure S1B, TRβ expression also increased *NCoR* expression in MCF7 cells, while significantly reducing *VEGF-C* and *VEGF-D* mRNA levels. NCoR deletion also enhanced considerably *VEGF-C* and *VEGF-D* transcripts in MCF-7 cells and reversed the repressive effect of TRβ to a significant extent (Supplementary Figure S1C), while SMRT deletion did not affect expression of the lymphangiogenic genes (Supplementary Figure S1D). These results reproduce those obtained in MDA cells, indicating that NCoR, but not SMRT, silences *VEGF-C* and *VEGF-D* gene expression in breast cancer cells independently of the ER status, and that NCoR is an important element in TRβ-dependent repression of these genes.

### NCoR and TRβ inhibit tumor lymphangiogenesis

**Figure 4:**
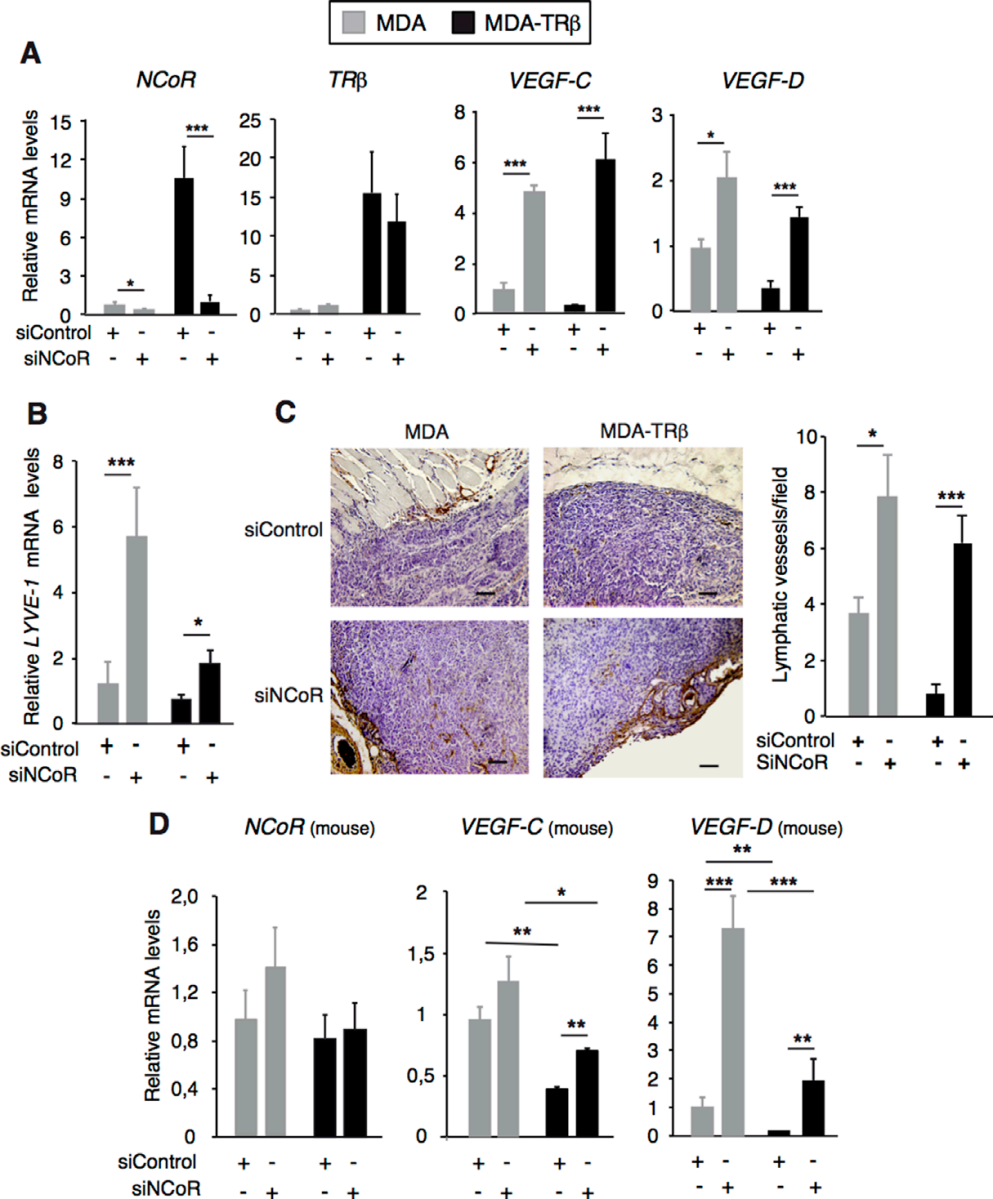
NCoR and TRβ inhibit tumor lymphangiogenesis (**A**) *NCoR*, *TRβ*, *VEGF-C* and *VEGF-D* mRNA levels in xenografts of MDA and MDA-TRβ cells. Cells were transfected with siControl or siNCoR 72 h before orthotopical inoculation into nude mice and animals were sacrificed 4 weeks later. (**B**) relative mouse LYVE-1 mRNA levels in the different groups of tumors. (**C**) representative immunohistochemical staining of LYVE-1 in the tumors, showing the presence of peritumoral lymphatic vessels. Bars: 100 μM.The right panel shows the quantification of the number of lymphatic vessels/microscopic field (means ± S.E) in the tumors. (**D**) mouse *NCoR*, *VEGF-C* and *VEGF-D* transcripts (means ± S.E) in the xenografts. mRNA levels (means ± S.E) are expressed relative to the values obtained in MDA tumors transfected with siControl. Statistically significant differences between tumors generated from breast cancer cells transfected with siControl and siNCoR, analyzed by ANOVA and Bonferroni pos*t*-test, are indicated as * *P* < 0.05, **P < 0.01 and ***P < 0.001.

To examine the effect of TRβ and NCoR in tumor lymphangiogenesis *in vivo*, we analyzed *VEGF-C* and *VEGF-D* gene expression as well as lymph vessel density in xenograft studies with MDA and MDA-TRβ cells transfected with siControl or siNCoR 72h before orthotopic inoculation into the fat mammary pad of nude mice. TRβ-expressing tumors were smaller and non invasive, but they became highly infiltrative in the absence of NCoR (Supplementary Figure S2). As expected from the existence of an autoregulatory loop that maintains *NCoR* gene expression [31], *NCoR* transcripts were still depleted in tumor xenografts formed by cells transfected with siNCoR more than one month before, while *TRβ* transcripts were not altered (Figure 4A). When *VEGF-C* and *VEGF-D* transcripts were measured, it was found that both genes were expressed al lower levels in tumors originated by MDA-TRβ cells than by the parental cells transfected with a control siRNA. However, NCoR depletion markedly enhanced lympangiogenic gene expression in both groups and under these conditions the repressive effect of TRβ on *VEGF-C* and *VEGF-D* expression was relieved, corroborating the results obtained in the cultured cells (Figure 4A). Transcript levels of mouse *LYVE-1* (or lymphatic vessel endothelial hyaluronan receptor), a specific marker of lymphatic vessels [41], was also reduced in the tumors formed by MDA-TRβ cells with respect to the MDA cells and NCoR depletion increased *LYVE-1* mRNA levels in parallel with the increased expression of the lymphangiogenic genes by the tumor cells (Figure 4B). Immunochemical detection of LYVE-1 showed that lymph vessels had a predominant peritumoral localization in the breast tumors formed by MDA cells and that they were very scarce in the tumors formed by the MDA-TRβ cells. However, vessel density increased significantly in NCoR-depleted tumors and the inhibitory effect of TRβ in tumor lymphangiogenesis was noticeably alleviated (Figure 4C).

Since not only the tumor cells but also the tumor microenvironment releases lymphangiogenic factors, we next examined mouse *VEGF-C* and *VEGF-D* expression in the xenografts (Figure 4D). Mouse NCoR levels were not altered by the human siRNA, showing the specificity of NCoR depletion exclusively in the tumor cells. However, host *VEGF-C* and *VEGF-D* expression was reduced in MDA-TRβ xenografts and increased when NCoR was depleted in the tumor cells. This indicates a reciprocal interplay between the tumor microenvironment and the tumor cells to collaboratively regulate tumor lymphangiogenesis.

Since TRβ expression reduces tumor size while tumors are bigger upon NCoR depletion (Supplementary Figure S2A), there was the possibility that the observed changes in lymphangiogenesis could be secondary to the different tumor size. Therefore, we next compared tumors formed by MDA and MDA-TRβ cells transfected with siControl or siNCoR when they reached the same volume (1 cm^3^). The results obtained confirmed again that NCoR depletion persisted for a very long time, since *NCoR* mRNA was significantly reduced both in MDA and MDA-TRβ cells originally transfected with siNCoR, whereas *VEGF-C* and *VEGF-D* transcripts were strongly induced, thus confirming the results obtained in the different size xenografts excised after one month (Supplementary Figure S3A). In addition, TRβ mRNA was not altered in the absence of NCoR, suggesting again that NCoR is the main mediator of the inhibitory effects of TRβ in lymphangiogenic gene repression. Moreover, *LYVE*-*1* gene expression followed a similar pattern (Supplementary Figure S3B), and mouse *VEGF-C* and *VEGF-D* mRNAs were induced by NCoR depletion (Supplementary Figure S3C), suggesting again that the absence of this corepressor induces changes in the tumor cells that affect the tumor microenvironment.

### NCoR depletion increases the presence of tumor DNA in the sentinel node

**Figure 5:**
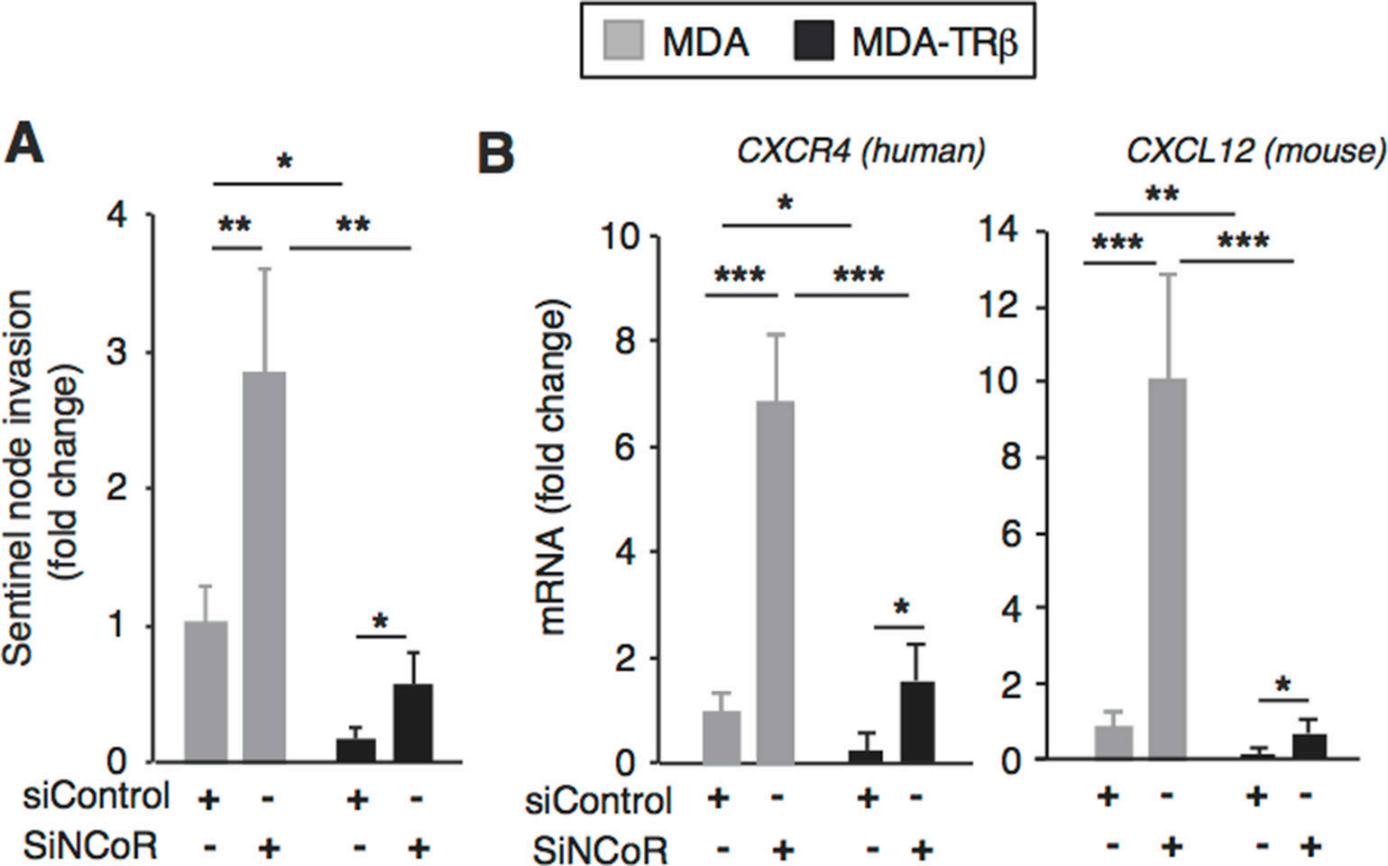
NCoR depletion increases chemokine expression and sentinel node invasion (**A**) sentinel lymph nodes were excised from the mice shown in Figure 4 that were inoculated 4 weeks before with MDA and MDA-TRβ cells previously transfected with siControl or siNCoR. The presence of the breast cancer DNA in the mice lymph node was assessed by determination of human *Alu* sequences. (**B**) transcript levels of the human *CXCR4* chemokine receptor and of the mouse *CXCL12* chemokine, its ligand, in the tumors. Data (means ± S.E) are expressed relative to the values obtained in mice injected with the parental cells transfected with siControl. Statistically significant differences were analyzed by ANOVA followed by Bonferroni test and are shown as * *P* < 0.05, **P < 0.01 and ***P < 0.001.

As expression levels of the lymphangiogenic factors correlate with lymph node metastasis, the sentinel lymph nodes of the mice were dissected and the presence of breast tumor DNA was studied by means of quantification of human *Alu* sequences. As shown in Figure 5A, in parallel with the changes in *VEGF-C* and *VEGF-D* expression, the presence of tumor DNA in the sentinel node was decreased in animals inoculated with TR ®-expressing cells, while NCoR depletion resulted in a significant increase in the amount of tumor DNA reaching the node and in a partial reversion of the inhibitory effect of TR ®.

The lymphatic endothelial cells produce chemokines that can attract the tumor cells expressing their receptors, supporting their migration toward the lymphatic vessels. Interestingly, it has been previously shown that CXCR4 is an NCoR and TRβ target gene [31, 38]. Therefore, we next analyzed expression of this receptor and its ligand in the tumors. Confirming previous results, *CXCR4* expression was reduced in the TRβ-expressing tumors and NCoR depletion resulted in a significant increase in its expression (Figure 5B). Remarkably, mouse *CXCL12* gene expression followed a similar pattern (Figure 5B), suggesting again the importance of the cross-talk between the breast cancer cells and the cells in the microenvironment in the regulation of tumor spreading.

### Correlation of NCoR and TRβ with lymphangiogenic gene expression in human breast tumors

**Figure 6:**
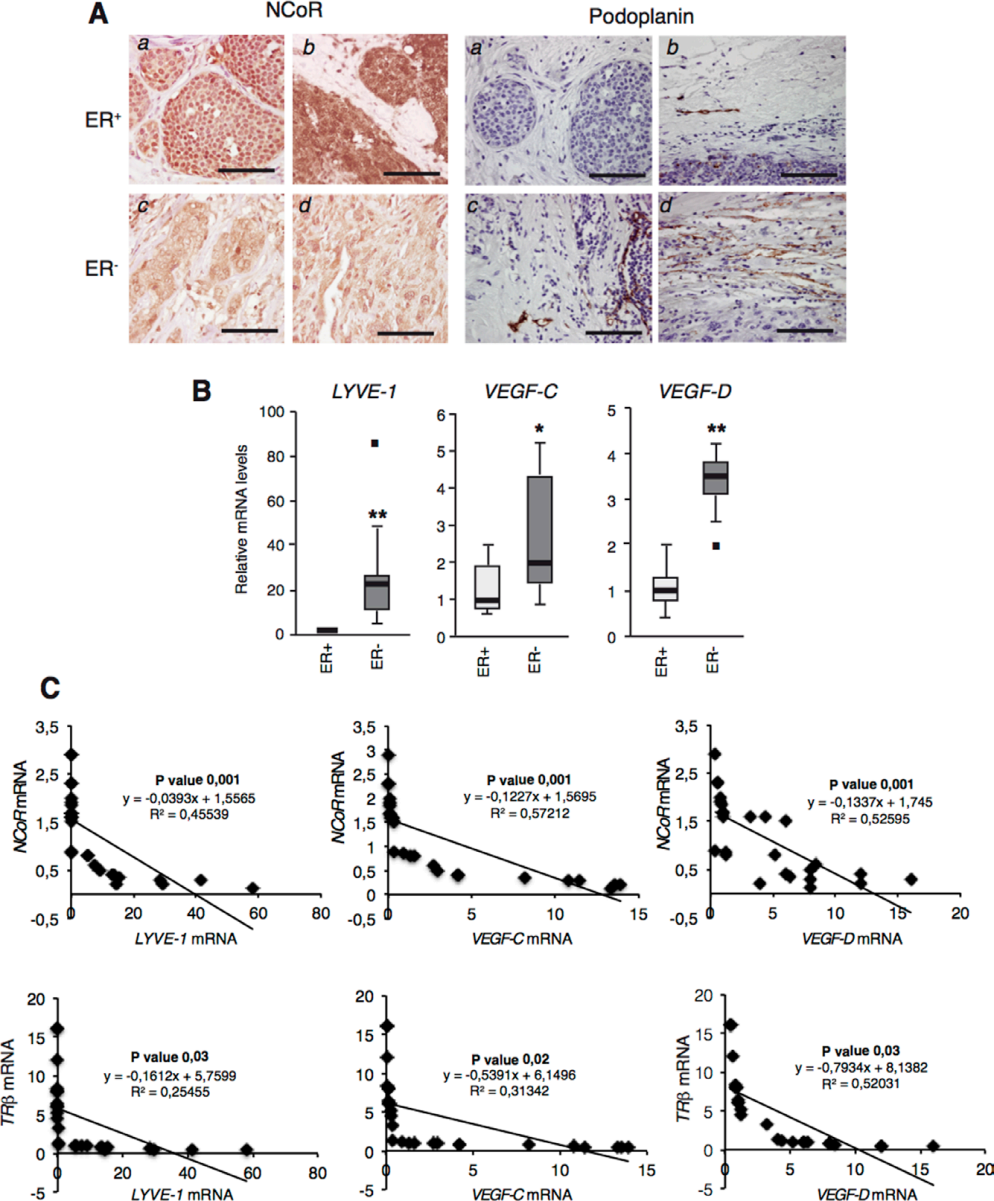
NCoR and TRβ levels correlate negatively with lymphangiogenic gene expresion in human breast tumors (**A**) NCoR (left panels) and Podoplanin (right panels) immunohistochemistry of representative ER^+^ and ER^−^ tumors. *a:* ER^+^ lobular tumor, *b* ER^+^ ductal tumor; *c* and *d* : ER^−^ ductal tumors. Bars: 100 μM. (**B**) Whisker plot of *LYVE-1*, *VEGF-C* and *VEGF-D* mRNA levels in ER^+^ and ER^−^ tumors. Data are mean ± S.E of 12 and 14 tumors, respectively. Outliers are shown by a black square. Significance of *t*-test between ER positive and negative tumors are indicated as * *P* < 0.05, **P < 0.01. (**C**) *NCoR* mRNA levels measured in Ref. were plotted against the corresponding *LYVE-1*, *VEGF-C* and *VEGF-D* mRNAs obtained in each sample. The *p* value and linear regression coefficient obtained are shown. (D) results with individual *TRβ* mRNAs obtained in Ref. 31, plotted against mRNAs of the lymphangiogenic genes.

To examine whether or not the repressive role of NCoR in lymphangiogenesis could be also demonstrated in human tumors, we next performed immunohistochemical staining of NCoR and lymphatic vessels in samples from 6 ER^+^ and 5 ER^−^ breast tumors. Figure 6A shows that NCoR staining was stronger in ER^+^ tumors than in ER^−^ tumors in which the majority of the cells did not show nuclear staining with the NCoR antibody. These results confirm previous data with other tumor series in which transcript levels of NCoR were also reduced in ER^−^ tumors [31]. In contrast, staining with the lymphatic vessel marker Podoplanin showed an opposite pattern with an increased number of peritumoral lymphatic vessels in the ER^−^tumors, suggesting that NCoR might also inhibit lymphangiogenesis in human breast tumors.

It has been shown that not only *NCoR* but also *TRβ* transcripts were markedly reduced in RNA samples from ER^−^ tumors when compared with ER^+^ tumors [31]. To further explore the potential role of these molecules in tumor lymphangiogenesis, we next quantitated *LYVE-1*, VEGF-C and *VEGF-D* transcripts in the same tumor series finding that, as expected, expression of these genes was higher in the more aggressive ER^−^ breast tumors (Figure 6B). To examine the possible existence of a negative correlation between NCoR or *TRβ* and lymphangiogenic gene expression, *NCoR* (Figure 6C) and *TRβ* (Figure 6D) mRNA levels were plotted against *LYVE-1*, *VEGF-*C and *VEGF-D* mRNAs. Statistical analysis showed that, indeed, there was a statistically significant negative correlation in all cases. The inverse relationship between transcript levels of *NCoR* and these genes was also found when ER^+^ and ER^−^ tumors were considered separately (Supplementary Figure S4), and this also occurred when *LYVE-1, VEGF-C* and *VEGF-D* mRNAs were plotted against *TRβ* mRNA levels (Supplementary Figure S5). These results further indicate that NCoR is also a potent inhibitor of lympangiogenesis in human breast tumors and a downstream effector of TRβ in this process.

## DISCUSSION

Studies in lymphangiogenesis have shown the key role of two members of the VEGF family, VEGF-C and VEGF-D, which interact with the VEGFR-3 receptor not only in the development of the lymphatic system but also in promoting tumor lymphangiogenesis and lymphatic metastasis [2, 11]. Therefore, identification of the mechanisms that regulate expression of these genes may be important to understand the molecular basis of lymphatic vessel growth and for the potential development of novel therapeutic strategies for combating metastasis. Breast tumors are particularly interesting at this respect, since spreading through the lymphatic system is predominant in these tumors. In the present study, we show that the corepressor NCoR and the nuclear receptor TRβ can inhibit transcription of the *VEGF-C* and *VEGF-D* genes, acting as potent repressors of tumor lymphangiogenesis in breast cancer xenograft models and correlating negatively with the expression of lymphangiogenic genes in human breast tumors.

The following findings clearly show that *VEGF-C* and *VEGF-D* are bona fide target genes for NCoR repression: i, breast cancer cells expressing higher NCoR mRNA levels express lower levels of the lymphangiogenic genes; ii, NCoR associates with the regulatory region of these genes in ChIP assays and this association is stronger in cells presenting higher levels of the corepressor; iii, NCoR depletion with siRNA increases promoter activity of the *VEGF-C* gene in transient transfection studies and iv, transfection with NCoR siRNA increases endogenous transcript levels of *VEGF-C* and *VEGF-D*. In addition to NCoR, TRβ also represses expression of lymphangiogenic genes, as indicated by the finding that *VEGF-C* and *VEGF-D*transcripts are significantly lower in breast cancer cell lines stably expressing TRβ than in their corresponding parental cells that express very low receptor levels. The silencing effect of TRβ appears to be mediated, at least in part, by the elevated NCoR levels present in TRβ expressing cells. This is proved by the result that lymphangiogenic transcripts are significantly restored upon NCoR depletion. However, at least in the case of *VEGF-C*, TRβ is recruited to the same promoter region responsible for NCoR binding and could therefore directly down-regulate transcription. In the case of *VEGF-D* NCoR, but not TRβ, associates with proximal promoter sequences. Interestingly, in breast cancer cells neither TRβ nor NCoR bind to a region previously reported to mediate regulation by some orphan receptors and to be important for basal transcription [40].

The role of NCoR and TRβ as potent regulators of the *VEGF-C* and *VEGF-D* genes was confirmed *in vivo* using xenografts. Thus, TRβ expression resulted in reduced transcript levels of these genes in the tumors, while NCoR depletion had an opposite effect enhancing them significantly. Again, the suppressive effect of TRβ was significantly reversed in the absence of NCoR, reinforcing the idea that NCoR plays a critical role in lymphangiogenic gene silencing by the receptor. Moreover, although tumor size is an important parameter to predict lymph node involvement in breast cancer [42], this occurred independently of tumor size, showing that direct regulation of lymphangiogenic gene expression is not secondary to the differences in tumor growth caused by TRβ or NCoR. These results suggested to us that these molecules might also suppress tumor lymphangiogenesis. Accordingly, histologic analysis showed that NCoR depletion was sufficient for promoting tumor lymphangiogenesis, resulting in increased *LYVE-1* gene expression and in increased density of LYVE-1–positive lymphatic vessels. Contrarily, TRβ-expressing tumors showed significantly reduced *LYVE-1* gene expression and a very low number of lymphatic vessels, which reappeared when NCoR was knocked-down. These lymphatic vessels were mainly in the peritumoral area. While intratumoral vessels are normally considered as nonfunctional, the lymphatic vessels at the periphery of the tumor likely serve as conduits for the metastatic cells to reach the draining lymph nodes. This has led to the concept that a dense lymphatic vasculature in this area would increase the number of entry sites of the tumor cells to the vessels and consequently metastatic spreading [43]. Accordingly, we found an increased amount of tumor DNA reaching the sentinel node upon NCoR depletion in the tumors and a much lower amount, also partially reversed in the absence of the corepressor, when the tumor cells expressed TRβ. These changes are compatible with parallel alterations in the number of metastatic cells colonizing the draining nodes, but the possibility that metastatic lesions were still not present and that DNA travelled to the lymph nodes in exosomes or by other means cannot be dismissed. Once the metastatic cells reach the lymph node they may enter a latent stage or further disseminate to other lymph nodes, the blood vessels and more distant organs.

The tumor microenvironment, composed by endothelial cells, immune cells, fibroblasts, signaling molecules and the extracellular matrix provides signals to the tumor cells in the form of both cell-cell contacts and secreted factors. It is increasingly evident that crosstalk between cancer cells and cells of the neoplastic microenvironment is a crucial component of invasion and metastatic growth [44]. Particularly interesting are the interactions between lymphatic endothelial cells and tumor cells to promote cancer cells dissemination. Not only the tumor cells secrete VEGFs, but also the tumor environment and specifically tumor-associated macrophages can function as a second source of lymphangiogenic factors [45]. Of interest, we found that expression of mouse *VEGF-C* and *VEGF-D* transcripts is increased in the tumors generated by NCoR-deficient breast cancer cells. As mouse NCoR gene expression is not altered, this suggests that breast tumor cells with different NCoR levels send distinct, still unidentified, signals to the macrophages that are able to alter lymphangiogenic gene expression. On the other hand, tumor cells may activate lymphatic cells to secrete factors that help their transport into the lymphatic vessels. Among them, lymphatic endothelial cells secrete chemokines such as CXCL12 that can promote tumor cells expressing the cognate receptor CXCR4 to migrate toward the lymphatic vessels, promoting a lymphatic microenvironment that supports tumor growth [19]. We have confirmed that CXCR4, a marker and mediator of breast-cancer metastasis [18, 46, 47], is a target for repression by NCoR and TRβ [31, 38]. Importantly, our results indicate that NCoR and TRβ not only silence the expression of CXCR4 receptors in the breast tumor cells, but also reduce the production of its ligand, CXCL12, by the tumor environment. The CXCL12-CXCR4 chemokine pathway has been shown to enhance tumor lymphangiogenesis and to have additive effects with the VEGF-C pathway. Furthermore, targeting both the chemokine and VEGF-C results in a stronger inhibition of tumor lymphangiogenesis and lymph node metastasis in the breast cancer xenograft model used in our study [48]. Therefore, the increased expression of these genes in the absence of NCoR would create a highly favorable scenario for lymphatic dissemination of breast cancer cells.

Besides silencing of VEGFs and chemokine receptors, we cannot exclude the possibility that NCoR and the receptor might also have additional anti-lymphangiogenic effects via modulation of other genes. Thus, in addition to VEGFs, a growing number of additional factors including IGFs [49], HGF [50] and COX2 [51, 52] have been described to promote lymphangiogenesis. Interestingly, the HGF receptor *c-Met* or *COX2* genes are TRβ and NCoR targets and the receptor induces the expression of *IGF-BP3*, an inhibitor of IGFs signaling [31, 38]. Therefore, it is likely that regulation of these factors might contribute to the anti-lymphangiogenic effects of TRβ and NCoR.

Lymphatic vessel density correlates with metastasis and poor outcome in most clinical studies in breast cancer and other tumors [3, 7, 8, 12, 14]. Accordingly, we found a higher number of lymphatic vessels in the more aggressive tumors. Furthermore, not only *LYVE-1* mRNA but also *VEGF-C* and *VEGF-D* mRNAs were lower in ER^+^ tumors. It had been previously shown that *NCoR* gene expression was reduced in the ER^−^ tumors [31] and, remarkably, there was a significant negative correlation, independent of the ER status, between *NCoR* and lymphangiogenic gene expression further validating that the corepressor is an important suppressor of lymphangiogenic genes transcription. A significant negative correlation between *VEGF-C* and *VEGF-D* and *TRβ* transcript levels was also found. This finding reinforces the hypothesis that TRβ is an upstream regulator of NCoR and suggests that the inhibitory effects of TRβ in human breast tumor lymphangiogenesis are most likely mediated by NCoR induction. These results suggest that NCoR and TRβ might be useful as novel biomarkers in breast cancer and as potential predictors of lymphatic dissemination. The development of therapeutic agents targeting lymphangiogenesis is being considered for the control of tumor growth and lymphatic metastasis. Thus, both the receptor and the corepressor might be novel therapeutic targets in both ER-positive and ER-negative tumors.

## MATERIALS AND METHODS

Extended materials and methods are provided in Supplementary Materials and Methods. Animal and human studies were approved by the Ethics Committee of the Consejo Superior de Investigaciones Científicas. NCoR and SMRT were knocked-down in cells with specific siRNA SMART pools from Dharmacon. Experimental procedures for transfections, luciferase reporter assays, western blot, mRNA determination by real time PCR and chromatin immunoprecipitation assays have been published previously and are described together with the antibodies and primers used in Supplementary Materials and Methods. Tumor formation in nude mice was followed for 4 weeks after orthotopic Supplementary Materials and Methods inoculation into the mammary pad. Histology and immunohistochemistry was performed by standard procedures. Significance of ANOVA pos*t*-test or the Student *t*-test among the experimental groups indicated in the figures is shown as *P < 0.05, **P < 0.01 and ***P <0.001.

## SUPPLEMENTARY MATERIALS


