# Supplementary material for: The nuclear corepressor 1 and the thyroid hormone receptor β suppress breast tumor lymphangiogenesis
Source: Oncotarget. 2016 Oct 27;7(48):78971–84. doi: 10.18632/oncotarget.12978 (PMC5346691; doi:10.18632/oncotarget.12978)
Supplement: Supplementary file 1 [file oncotarget-07-78971-s001.pdf]

The nuclear corepressor 1 and the thyroid hormone receptor β suppress breast tumor lymphangiogenesis

Supplementary Materials

Study approval

Animal experiments were carried out in the animal facility of the Instituto de Investigaciones Biomédicas in compliance with the European Community Law (86/609/EEC) and the Spanish law (R.D. 1201/2005), with approval of the Ethics Committee of the Consejo Superior de Investigaciones Científicas. Animals were always randomly distributed among the experimental groups. Use of human breast cancer RNA and paraffin samples was performed following standard ethical procedures of the Spanish regulation (Ley Orgánica de Investigación Biomédica, 14 July 2007) and has been approved by the Ethics Committee of Clinical Investigations of the Ramón and Cajal Hospital and the Ethics Committee of the Consejo Superior de Investigaciones Científicas. Paraffin samples and data from patients included in this study (Supplementary Table 1) were provided by the Hospital Universitario Ramón y Cajal-IRYCIS Biobank integrated in the Spanish Hospital Platform Biobanks Network (RetBioH; www.redbiobancos.es) and were processed following standard operation procedures with appropriate approval of the Ethical and Scientific Committees. RNA samples from breast tumors used in a previous study[31] were obtained from the HUVR-IBiS Biobank (Andalusian Public Health System Biobank and ISCIII-Red de Biobancos PT13/0010/0056).

Cell lines

MDA-MB-231 and MCF-7 cells were grown with DMEM and HCC1954 and ZR75-1 cells with RPMI containing 10% FBS. Cell lines were authenticated analyzing STRs of specific loci of the Human Genome with the StemElite ID system (Promega). Cells were also tested regularly for mycoplasma contamination and all cells used were negative for mycoplasma. MDA-MB-231 cells were infected with supernatants of HEK293 cells transduced with the pLPCX vector or with the retroviral vector pLPCX-TRβ encoding the human TRβ1 receptor as previously described [38]. Cells were selected with 2 mg/ml puromycin and pools of resistant cells (hereafter named MDA and MDA-TRβ cells, respectively) were cultured in 10% FBS depleted of thyroid hormones by treatment with resin AG-1-X8 (Bio-Rad). Parental MCF-7 cells as well as cells expressing TRβ1[37] were a kind gift from S-Y Cheng.

Nude mouse xenograft model

Mice were housed in pathogen-free condition, in a 12/12 h light/dark cycle with water and normal diet food available ad libitum. Animals were always randomly distributed among the experimental groups. Following ethical approval, 2 million cells transfected 72 h before with a control siRNA or with a NCoR specific siRNA were injected ortothopically into the fat pad of the second abdominal mammary gland of female nu/nu mice, aged 6–8 weeks, and allowed to form tumors. Tumor volume (12–14 tumors/group) was measured once a week with a caliper and mice of all groups were sacrificed 4 weeks after inoculation. The tumors were collected and sentinel lymph nodes were removed for DNA extraction. The tumors were split, a portion was fixed in 4% formalin and embedded in paraffin blocks for histology and immunohistochemistry (IHC), and the remainder was kept frozen for protein and mRNA determination. In other experiment, 1 million cells were inoculated orthotopically after the same treatments (3 tumors/group), and animals were sacrificed when the tumors reached 1cm^3.

siRNA knockdown

Validated siRNA SMART pools targeted towards human NCoR (M-058556) and SMRT (M-045364) were purchased from Dharmacon. These pools have been shown to be specific and to lack off-target effects[31]. Cells were transfected with 33 ng of NCoR siRNA (siNCoR), SMRT siRNA (siSMRT), or with non-specific control pool (siControl) (M-001210) using Lipofectamine 2000 (Invitrogen) following the manufacturer's instructions. After transfection, cells were cultured for 72 h in the presence or absence of 5 nM T3.

Histology and Immunohistochemistry. For histopathologic procedures tumors were fixed in 4% buffered formalin and embedded in paraffin wax

Deparaffinized sections (4-5 μm) were stained with H&E using standard procedures. Images were obtained using a high-resolution Leica DC200 digital camera mounted on an Olympus DMLB microscope. Immunohistochemistry with LYVE-1 (ab-36993),

Podoplanin (Dako D2-40) and NCoR (sc-8994) antibodies was performed on 4µm deparaffinized-rehydrated sections. In the human tumors, lymphatic vessels were detected in Podoplanin-stained slides, since labeling with the LYVE-1 antibody was very unspecific. Antigen retrieval was carried out with citrate buffer and endogenous peroxidase activity was inhibited with 3% H_2O_2. Samples were blocked and incubated overnight with the antibodies, and signal was amplified with the ABC *Kit* (Vectastin). Slides were revealed with DAB (Vector), counterstained with Hematoxylin and mounted with DePeX (Serva). Lymphatic vessel density of the tumor xenografts was determined by scoring the number of LYVE-1 positive vessels/field in at least 10 fields by an observer blind to the experimental group of the mice.

Real-time quantitative RT-PCR (qRT-PCR)

Total RNA was extracted from cells using Trizol (Sigma) and from tumors using RNeasy Mini Kit (Quiagen). mRNA levels were analyzed in technical triplicates by quantitative RT-PCR, following specifications of SuperScript™ First-Strand Synthesis System (Invitrogen). Data analysis was done using the comparative CT method and data were corrected with GAPDH mRNA levels. Primers used for amplification of human and mouse transcripts are listed in the Supplemental material. No CTs were obtained when human transcripts were amplified with mouse oligonucleotides and vice versa, indicating species specificity.

Quantification of sentinel lymph node invasion

The presence of human breast cancer in the mice sentinel node was estimated by real-time PCR of human *APO Alu* sequences located in human chromosome 11 using the following primers: Forward: 5′-AAGTGCTGTAGGCCATTTAGATTAG-3′ and Reverse: 5′-AGTCTTCGATGACAGCGTATACAGA-3′. The % of amplified human DNA, with respect to the amount of total DNA injected originally into the mammary gland, was calculated. No amplification of human *Alu* sequences was found in tissues of mice that were not inoculated with human breast cancer cells that were used as negative controls.

Transfection and reporter assays

Cells were transiently transfected with 300 ng of reporter plasmids containing the 5′-flanking region of the human VEGF-C gene[39] and 30 ng of pRL-TK-Renilla (Promega) as a normalizer control, using Lipofectamine 2000. When appropriate, the reporter was cotransfected with 33 ng of control or NCoR siRNAs. Luciferase activity was measured in untreated cells and in cells treated with 5 nM T3 for 36h using the Dual Luciferase Assay System (Promega). Each experiment was performed in triplicate and was normally repeated at least 3 times.

Western blotting

Proteins from cell lysates (80µg) were separated in SDS-PAGE and transferred to nitrocellulose membranes (Protran Whatman, Perkin Elmer) that were blocked for 2 h at room temperature with 4% BSA or non-fat milk. Incubation with primary antibodies was performed overnight at 4ºC and with the secondary antibody for 1 h at room temperature. Blots were visualized with ECL (Amersham). The antibodies used are listed in the Supplementary material.

Chromatin immunoprecipitation

Cells were plated in 150mm dishes, fixed and lysed following specifications of the Upstate kit (cat. 17-295), and sonicated in a Bioruptor UCD-200TM (Diagenode). For each immunoprecipitation. 2-3x10^6 cells and 5 µg of NCoR (sc-8994) or 5 µg TRβ (sc-737) antibodies were used. The same amount of control IgG was used as a negative control. DNA was purified, precipitated and used for real-time PCR amplification of the 5′-flanking regions of the human *VEGF-C* and *VEGF-D* genes listed in the Supplementary material. Data are expressed as % of the input DNA in each reaction. The IgG values were always lower than 1% of the input and when indicated were subtracted from the total. Data shown were obtained in 2 independent experiments performed in three technical replicates.

Statistical analysis

Statistical significance of data was determined by applying a two-tailed Student *t*-test or ANOVA followed by the Bonferroni test for experiments with more than two experimental groups. Shapiro-Wilk test was used to check a normal distribution and Levene test to assess the equality of variances. Results obtained in animals are expressed as means ± s.e.m and in cultured cells as the mean ± s.d. Significance of ANOVA post-test or the Student *t*-test among the experimental groups indicated in the figures is shown as * $P<0.05$, ** $P<0.01$ and *** $P<0.001$. Linear regressions were calculated with the SPSS software. No previous calculations were used to determine sample size, which was chosen based on usual procedures in the field.

Primers used for quantitative PCR

Human

CXCR4

5′-ATCTTTGCCAACGTCAGT-3′ (forward)
5′-TCACACCCTTGCTTGATG-3′ (reverse)

L19

5′-GCGTGCTTCCTTGGTCTTAG-3′ (forward)
5′-AAGAAGATCGATCGCCACA-3′ (reverse)

LYVE-1

5′-ATCCCCTTACTCTACAATACCTGC-3′ (forward)
5′-GACATAGCAAAATCCAAGACCAG-3′ (reverse)

NCoR

5′-TAAGTCCTGCCAGCTACCT-3′ (forward)
5′-GGAGTCTGCGAGGAAACTTG-3′ (reverse)

SMRT

5′-TGTCACCTCAGCCAGCATAG-3′ (forward)
5′-CGCCGTAAGTAGTCCTCCTG-3′ (reverse

TRβ

5′-GAACAGTCGTCGCCACATC-3′ (forward)
5′-GCTCGTCCTTGTCTAAGTA-3′ (reverse)

VEGF-C

5′-CAGACAAGTTCATTCAATTATTAGACG-3′ (forward)
5′-CATGTCTTGTTAGCTGCCTGA-3′ (reverse)

VEGF-D

5′-CAACTTTCTATGACACTGAAACAC-3′ (forward)
5′-TCTCTCTAGGGCTGCATTGG-3′ (reverse)

Mouse

CXCL12

5′-CAGAGCCAACGTCAAGCA-3′ (forward)
AGGTACTCTTGGATCCAC-3′ (reverse)

LYVE-1

5′-CGACCTCGTGCAAGACCTTT-3′ (forward)
5′-GGAGTTAACCCAGGTGTCGG-3′ (reverse)

NCoR

5′-GCTGCAGGAGAGGTTTATCG-3′ (forward)
5′-CCTGCATCTGCTGTGAGGTA-3′ (reverse)

VEGF-C

5′-CAGACAAGTTCATTCAATTATTAGACG-3′ (forward)
5′-CATGTCTTGTTAGCTGCCTGA-3′ (reverse)

VEGF-D

5′-CAACTTTTCTATGACACTGAAACACA-3′ (forward)
5′-TCTCTCTAGGGCTGCATTGG-3′ (reverse)

GAPDH

5′-ACAGTCCATGCCATCACTC-3′ (forward)
5′-GCCTGCTTCACCACCTTCTT-3′ (reverse)

Primers used for ChIP assays

Human VEGF-C promoter

-235/+13
5′-GGCTTTTACCTGACACCCGC-3′ (forward)
5′-CCAGCAAGTGCATGGTGG-3′ (reverse)
-550/-232
5′-GGATCCTGCGCCGCGGCGC-3′ (forward)
5′-CCTCACAGGAAACCGGACATCCG-3′ (reverse)

Human VEGF-D promoter

-423/-119
5′-GTTTCAATCCAGAGTTGG-3′ (forward)
5′-GGAAAGAATCAGTTCTGATC-3′ (reverse)
-608/-430
5′-GTCATTGGCAGCAGATGCATG-3′ (forward)
5′-GCACAACCTTCATGGAAGCTTGC-3′ (reverse)

Antibodies used

IgG (5 μg in ChIP assays). Sc-2027
ERK (1:5000 in Western blot). Santa Cruz (sc-154).
Lamin β (1:1000 in Western blot). Santa Cruz (sc-6216).
LYVE-1 mice (1:500 in immunohistochemistry). Abcam (ab-14917).
Podoplanin human (1:400 in immunohistochemistry). Dako (D2-40)
NCoR (1:500 in Western blot, 1:50 in immunohistochemistry, 5 μg in ChIP assays). Santa Cruz (sc-8994).
SMRT (1:1000 in Western Blot(1:1000 in Western blot). Santa Cruz (sc-772).
TRβ (1:500 in Western blot, 5 μg in ChIP assays). Santa Cruz (sc-737).

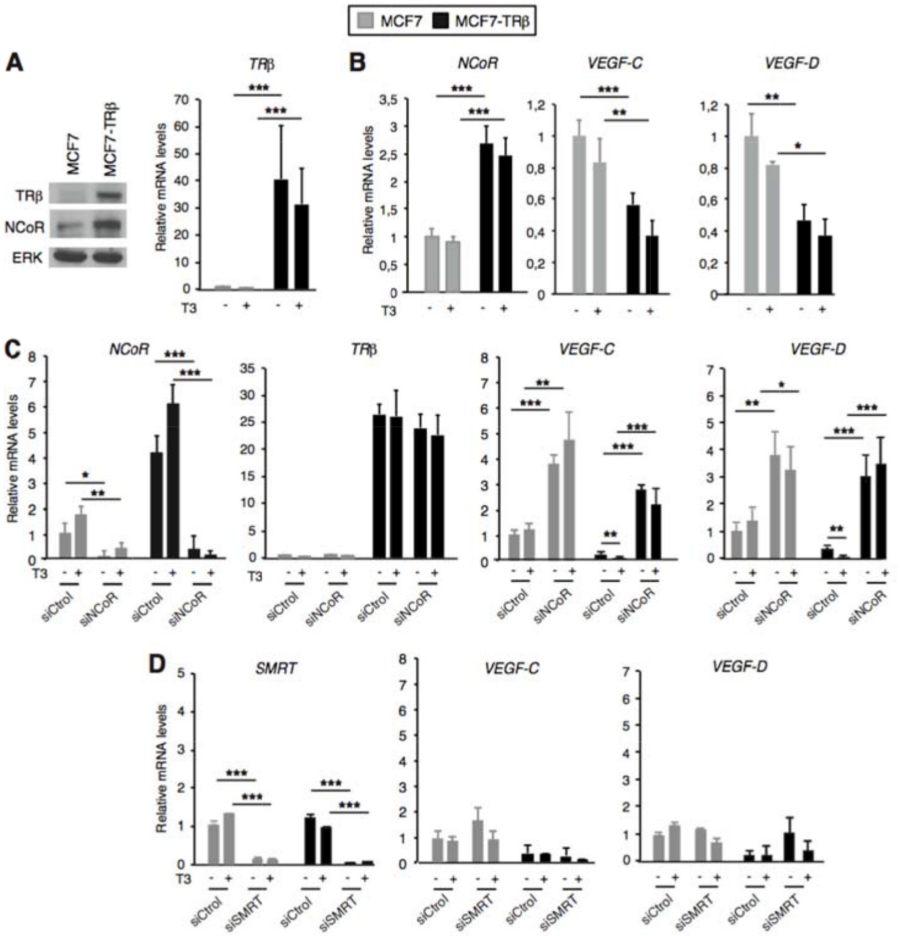

Supplementary Figure S1: NCoR but not SMRT depletion increases expression of lymphangiogenic genes in MCF-7 cells. (**A**) TRβ and NCoR protein and mRNA levels in MCF7 and MCF7-TRβ cells. (**B**) *NCoR*, *TRβ*, *VEGF-C* and *VEGF-D* transcripts (means ± SD) were determined in parental in TRβ-expressing cells incubated in the presence and absence of 5 nM T3 for 36 h, as indicated. (**C**) mRNA levels (means ± SD) of the indicated genes determined in cells transfected with Control or NCoR siRNAs and treated with and without T3. (**D)** similar experiment in cells transfected with Control or SMRT siRNAs. The existence of statistically significant differences after ANOVA post-test is indicated as $*P < 0.05$, $**P < 0.01$ and $***P < 0.001$.

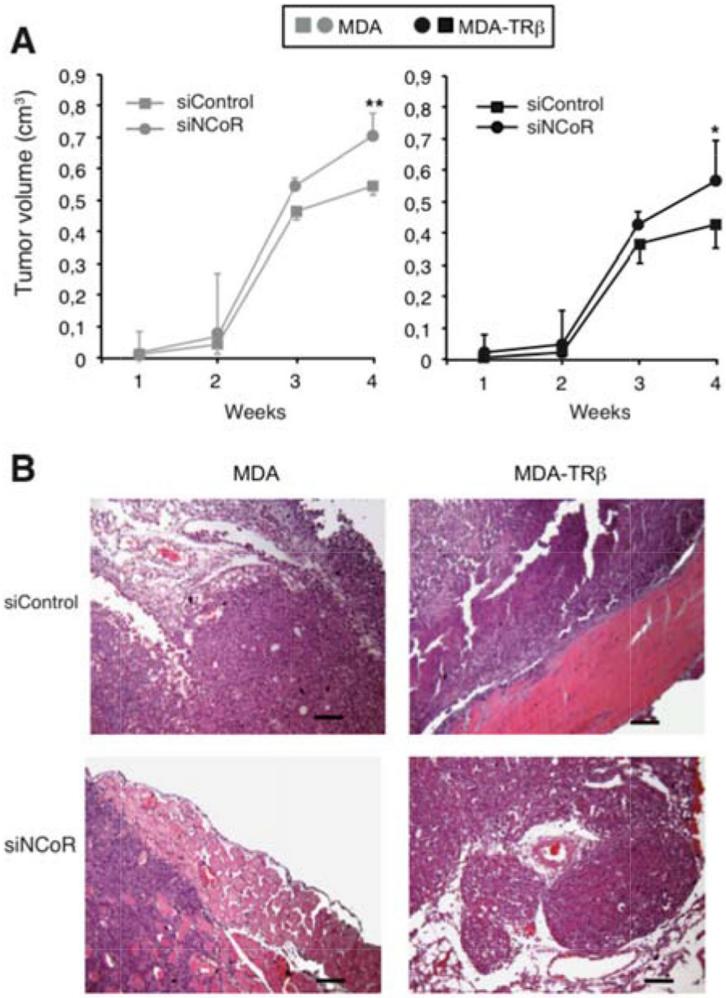

Supplementary Figure S2: TRβ reduces, while NCoR depletion increases, the growth and invasion of tumor xenografts formed by MDA breast cancer cells. (**A**) MDA and MDA-TRβ cells were transfected with siControl or siNCoR and 72 h later were inoculated into the fat mammary pad of immunodeficient mice. Volume of the tumors (mean ± S.E) was measured once a week until sacrifice at 4 weeks after inoculation. Asterisks denote the existence of statistically significant differences between xenografts formed by cells originally transfected with siControl and siNCoR. *$P < 0.05$, **$P < 0.01$. (**B**) representative H&E staining of the tumors from the different groups. Note that xenografts formed by MDA-TRβ cells are not invasive but they become invasive upon NCoR depletion.

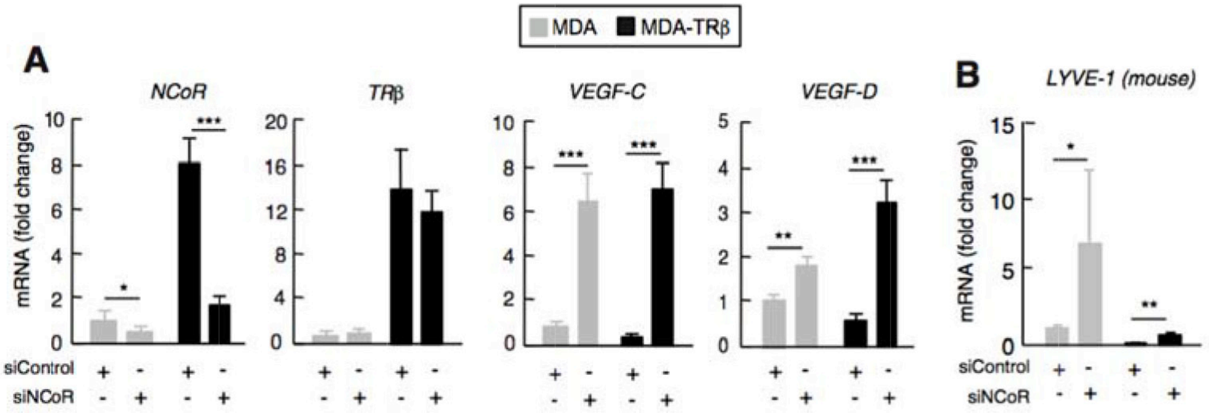

Supplementary Figure S3: Lymphangiogenic gene expression in MDA and MDA-TRβ tumors. (**A**) nude mice were inoculated orthotopically with MDA and MDA-TRβ cells transfected 3 days before with siControl or siNCoR and xenografts were dissected when they reached a volume of 1 cm^3. This size was reached at an average of 63 days with MDA cells transfected with siControl, 42 days with MDA cells transfected with siNCoR, 83 days with MDA-TRβ cells transfected with siControl and 56 days with MDA-TRβ cells transfected with siNCoR. Transcript levels (mean ± S.E) for the indicated human genes were determined in the tumors. (**B**) mouse LYVE-1 mRNA levels in the same tumors. (**C**) mouse *NCoR*, *VEGF-C* and *VEGF-D* transcripts (means ± S.E) in the xenografts. Statistically significant differences between values obtained in tumors originated from cells transfected with siControl and siNCoR are indicated as $*P < 0.05$, $**P < 0.01$ and $***P < 0.001$.

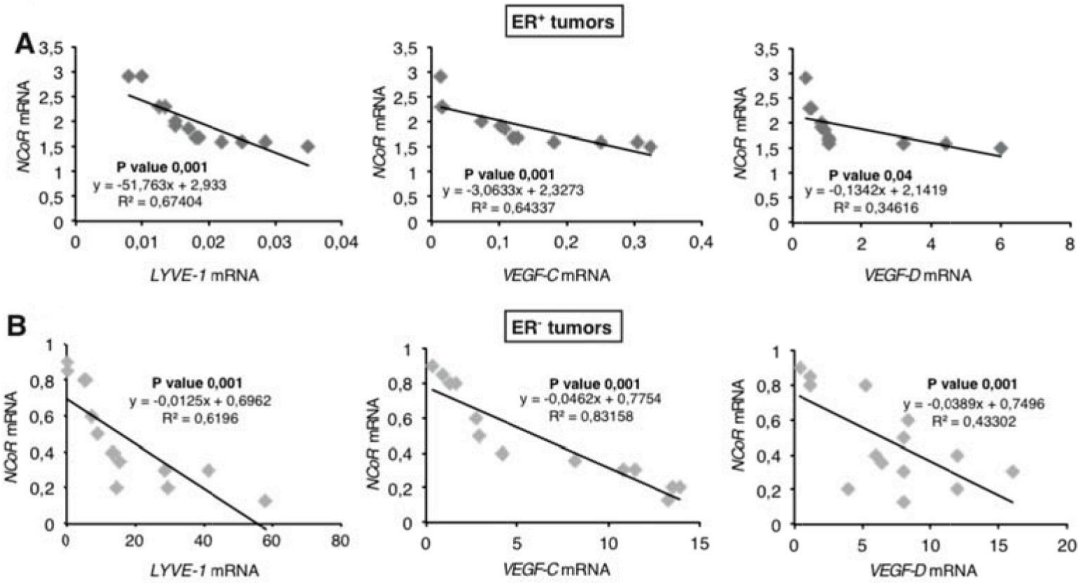

Supplementary Figure S4: Expression of lymphangiogenic genes correlates negatively with *NCoR* gene expression both in ER positive and ER negative human breast tumors. (**A**) individual values of *NCoR* mRNA were plotted against the corresponding *LYVE-1*, *VEGF-C* and *VEGF-D* mRNAs and regression coefficients and p values were calculated for ER^+ tumors ($n = 12$). (**B**) similar analysis for the ER^- tumors ($n = 14$).

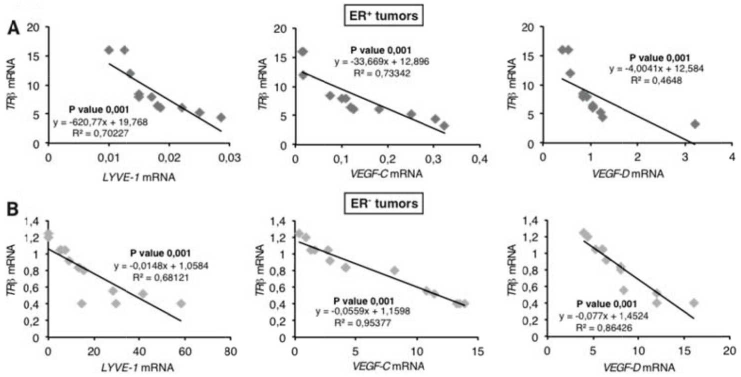

Supplementary Figure S5: Negative correlation of *TRβ* and lymphangiogenic gene expression in both ER positive and ER negative human breast tumors. (**A**) individual values of *TRβ* mRNA were plotted against the corresponding *LYVE-1, VEGF-C* and *VEGF-D* mRNAs and regression coefficients and *p* values were calculated for ER^+ tumors. (**B**) similar analysis for the ER^- tumors.

Supplementary Table S1: Clinical and pathological data of the human breast tumors used for immunohistochemistry

PATIENT #	ER STATUS	DIAGNOSIS
1	ER+	Infiltrating lobular carcinoma
2	ER+	Infiltrating ductal carcinoma
3	ER+	Infiltrating ductal carcinoma
4	ER+	Infiltrating carcinoma
5	ER−	Infiltrating ductal carcinoma
6	ER−	Infiltrating ductal carcinoma
7	ER−	Infiltrating ductal carcinoma
8	ER+	Infiltrating ductal carcinoma
9	ER+	Infiltrating lobular carcinoma
10	ER−	Infiltrating ductal carcinoma
11	ER−	Infiltrating ductal carcinoma
